# Pulmonary injury in inflammatory bowel disease: intestinal barrier disruption, gut–lung axis remodeling, and treatment-related lung toxicity

**DOI:** 10.3389/fimmu.2026.1883855

**Published:** 2026-07-14

**Authors:** Ziyi Mu, Renmin Zhou, Wujuan Hao, Ying Chen, Xingli Zhu, Lan Gu, Qiong Lin

**Affiliations:** 1 Affiliated Children’s Hospital of Jiangnan University (Wuxi Children’s Hospital), Wuxi, Jiangsu, China; 2 Department of Gastroenterology, Affiliated Children’s Hospital of Jiangnan University (Wuxi Children’s Hospital), Wuxi, Jiangsu, China

**Keywords:** inflammatory bowel disease, lung injury, intestinal mucosal barrier, gut–lung axis, short-chain fatty acids, drug-induced lung injury

## Abstract

Inflammatory bowel disease (IBD)-associated pulmonary injury is one of the frequently underestimated extraintestinal manifestations in clinical practice. It can present as subclinical pulmonary function abnormalities, radiographic changes, or overt inflammatory pulmonary diseases and often overlaps with infection- and treatment-related pulmonary toxicity, complicating early recognition and differential diagnosis. Growing evidence indicates that IBD-associated pulmonary injury is not an isolated pulmonary event but rather a cross-organ pathological process jointly driven by disruption of the intestinal mucosal barrier, dysbiosis, and aberrant microbial metabolites, systemic inflammation, and abnormal immune cell trafficking. This article provides a comprehensive review of current basic and clinical research evidence regarding intestinal barrier dysfunction, bidirectional gut–lung axis regulation, immune remodeling mediated by microbial metabolites, such as short-chain fatty acids (SCFAs), and the mechanisms and differential diagnosis of drug-induced pulmonary injury in IBD treatment. Existing studies suggest that SCFAs, tryptophan metabolites, immune cell homing, and alterations in the local pulmonary microbiota may represent key regulatory nodes in IBD-associated pulmonary injury; however, prospective studies integrating intestinal inflammatory activity, circulating biomarkers, pulmonary phenotypes, and drug exposure are still lacking. In the future, emphasis should be placed on establishing a stratified diagnostic framework for distinguishing pulmonary involvement intrinsic to the disease, infection, and drug-induced pulmonary injury, and on evaluating precision intervention strategies targeting microbial function, metabolite supplementation, and barrier repair to advance the translational application of mechanistic research on IBD-associated pulmonary injury into clinical practice.

## 1 Introduction

Inflammatory bowel disease (IBD) refers to a group of chronic, relapsing, non-infectious inflammatory disorders of the intestine, encompassing two main types: Crohn’s disease (CD) and ulcerative colitis (UC) (1, 2). In recent years, the incidence and prevalence of IBD have continued to rise worldwide, with an increasing trend among younger populations (3). IBD develops during childhood in approximately 10%–20% of cases (4). However, the exact etiology remains incompletely elucidated. Current evidence indicates that the pathogenesis of IBD involves a complex interplay of multiple factors, including genetic susceptibility, mucosal barrier dysfunction, gut microbiota dysbiosis, and aberrant immune activation (1–3). Clinically, IBD is characterized predominantly by diarrhea, abdominal pain, mucus- and blood-streaked stools, and weight loss; in severe cases, complications such as intestinal obstruction and fistula formation may occur, suggesting its essence as a progressive pathological process driven by systemic immune homeostasis disruption (1). Notably, up to 24% of IBD patients develop extraintestinal manifestations (EIM) affecting multiple organs, including the joints, skin, eyes, hepatobiliary system, and respiratory tract, prior to the onset of intestinal symptoms, significantly impacting quality of life and long-term prognosis. Approximately a quarter of EIM may precede classic gastrointestinal complaints, resulting in delays in early recognition and diagnostic blind spots (5). Among the various EIM, pulmonary involvement has long been underestimated: although most patients lack typical respiratory symptoms, pulmonary function tests (PFTs) or chest imaging commonly reveal abnormal changes (6). Studies have found a high proportion of subclinical pulmonary abnormalities in IBD populations, commonly manifesting as decreased diffusing capacity of the lung for carbon monoxide (DLCO), reduced forced expiratory volume in one second (FEV1), small airway impairment, and high-resolution computed tomography (HRCT) findings such as centrilobular nodules and bronchial wall thickening (6–8). Among these pulmonary function abnormalities, reduced DLCO appears to be relatively common in pediatric patients with IBD (9). Ateş et al. found that among IBD patients without overt respiratory symptoms, abnormal pulmonary function parameters correlated with disease activity (7). Sato et al., in an HRCT evaluation of 601 IBD patients, showed that both UC and CD can present a high prevalence of imaging abnormalities, predominantly centrilobular nodules and bronchial wall thickening, with some differences in radiological features between subtypes (8). These findings suggest that the hidden burden of IBD-associated lung injury may arise earlier than clinically overt respiratory disease, highlighting the importance of detecting early changes and elucidating mechanisms for timely intervention (6, 8).

The anatomical basis of IBD-associated lung injury can be traced back to organ embryogenesis. The gut and lung share an endodermal developmental origin, with the respiratory epithelium arising from the anterior foregut endoderm (10). This ontogenetic relationship is reflected in shared mucosal features, including epithelial barrier specialization, mucus-producing cells, and mucosa-associated lymphoid tissue (MALT), which may facilitate coordinated immune responses across the gut–lung axis (11). Based on this developmental homology, the gut and lungs form an integrated common mucosal immune system (CMIS). Studies have shown that intestinal antigen-activated immune cells, including lymphocytes expressing C-C chemokine receptor type 9 (CCR9), may undergo cross-homing through the bloodstream and migrate to pulmonary tissues with shared mucosal characteristics (12). Therefore, in the context of IBD-induced intestinal barrier disruption and immune dysregulation, this developmentally established gut–lung axis becomes a conduit for the dissemination of inflammatory mediators and sensitized immune cells from the gut to the lungs, potentially triggering or aggravating pulmonary injury.

Within this theoretical framework, breakdown of the intestinal barrier represents the key initial step connecting intestinal inflammation to distal organ injury. The intestinal barrier comprises the mucus layer, specialized epithelial cells and their tight junctions (TJs), antimicrobial peptides/secretory immunoglobulin A (IgA), and the mucosal immune network. Its core function is to facilitate nutrient absorption while limiting microbial and antigen translocation from the gut lumen into the body (13). When inflammatory processes disrupt the structure and function of tight junctions, paracellular permeability increases, and pathogen-associated molecular patterns (PAMPs) and antigens more readily translocate across the mucosa, triggering sustained innate immune activation and driving the systemic spread of inflammation (14), ultimately manifesting as distal pulmonary phenotypes. Alterations in the expression and localization of claudin family proteins are recognized as fundamental molecular mechanisms contributing to the “leaky barrier” associated with IBD. Zeissig et al. demonstrated that the upregulation of pore-forming claudin-2, alongside the downregulation or mislocalization of sealing claudins, is closely linked to impaired barrier function in patients with active CD (15). Further evidence underscores that inflammatory mediators can modulate tight junction complexes and cytoskeletal pathways, thereby increasing epithelial permeability and establishing a vicious cycle of inflammation-driven barrier dysfunction (13, 16, 17). Importantly, this barrier-centered concept also raises a translational question: whether clinically measurable markers of intestinal permeability and microbial translocation can help connect mucosal barrier failure with systemic immune activation and pulmonary inflammatory consequences.

Meanwhile, gut microbiota and their metabolites provide an interpretable and modifiable molecular link for cross-organ immunoregulation. Short-chain fatty acids (SCFAs), produced by the fermentation of dietary fiber by gut microbiota, can regulate dendritic cell function, regulatory T cell (Treg) homeostasis, and the inflammatory threshold via mechanisms such as receptor-mediated signaling and histone deacetylase (HDAC) inhibition, and have been shown to affect the pulmonary immune environment and the severity of allergic airway inflammation (18–20). Trompette et al. provided experimental evidence for a dietary fiber-gut microbiota-SCFAs-lung immunity axis, suggesting that microbial metabolites can serve as key effector molecules in gut–lung axis communication (19). In addition to SCFAs, gut microbial tryptophan metabolites can activate the aryl hydrocarbon receptor (AhR), balancing mucosal reactivity and barrier repair via interleukin-22 (IL-22) and other pathways, thus providing another critical clue for explaining IBD-related mucosal immune imbalance and its distal effects (21). Beyond these two well-characterized metabolite classes, selected additional microbiota-modulated pathways, including bile acid remodeling, bacterial sphingolipid metabolism, succinate signaling, and the trimethylamine/trimethylamine N-oxide (TMA/TMAO) axis, may further extend the metabolic framework underlying gut–lung axis remodeling.

In addition to the route of metabolites entering the bloodstream, immune cell migration and intercellular communication networks may also mediate the dissemination of intestinal inflammation to the lungs. Under homeostatic conditions, intestinal mucosal immune cells express defined homing molecules, including integrin α4β7 integrin and CCR9, which help maintain gut-directed immune surveillance; however, under chronic inflammatory conditions, these homing programs may be remodeled, promoting the migration of effector cells to distal mucosal tissues and participating in local immune microenvironment reprogramming and amplification of tissue damage (20). Therefore, IBD-related pulmonary injury is likely to result from convergent pathways involving barrier disruption, microbiota/metabolite shifts, systemic inflammation, and immune migration/communication, rather than being confined to a single organ.

Notably, the clinical and radiological manifestations of IBD-related pulmonary injury often overlap with those of drug-related lung injury, significantly increasing diagnostic difficulty. With the widespread use of biologics and immunomodulators, the importance of pharmacovigilance for non-infectious drug-related pneumonitis/interstitial lung disease (drug-related pneumonitis/ILD) in the IBD population has become increasingly apparent (22, 23). Pugliese et al. reported a case series of vedolizumab-associated drug-induced pneumonitis, emphasizing the importance of early recognition and drug withdrawal (22). In addition, there are well-documented cases of interstitial pneumonia related to 5-aminosalicylic acid (5-ASA, such as mesalazine), which should be cautiously diagnosed only after ruling out infection and EIM-related pulmonary involvement (24). Eliadou et al. provided a summary analysis of cases of IBD with interstitial/granulomatous lung disease, further indicating that “pulmonary EIM from the disease itself” and “drug-induced lung injury” frequently coexist in the same clinical context, highlighting the urgent need to establish more feasible classification and diagnostic pathways (25).

**Figure 1:**
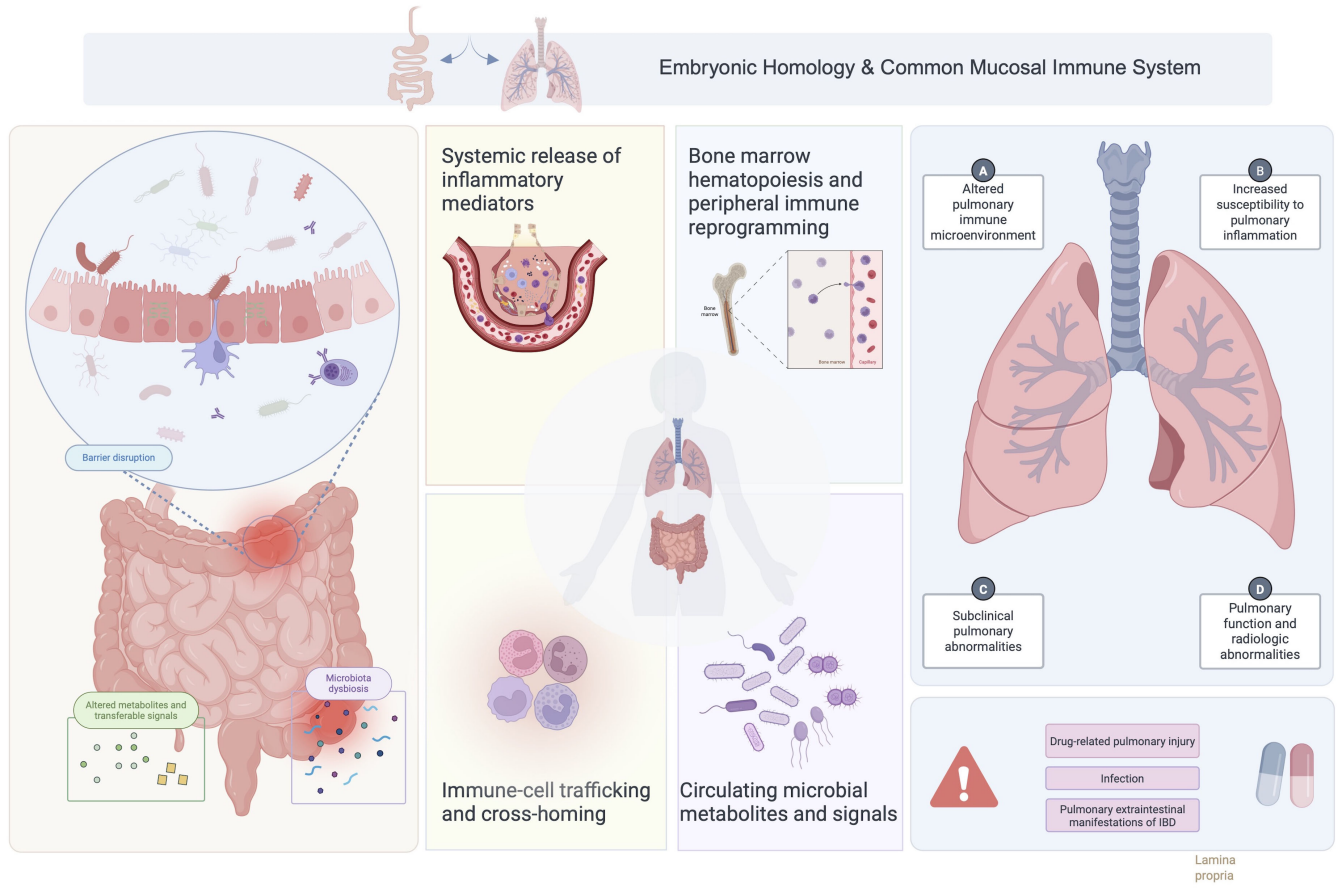
Proposed integrative framework for pulmonary injury in inflammatory bowel disease. Pulmonary injury in inflammatory bowel disease (IBD) may result from the convergence of multiple interconnected mechanisms rather than from an isolated pulmonary process. The gut and lungs are linked by embryonic homology and the CMIS, providing the biological basis for cross-organ immune crosstalk. In IBD, intestinal inflammation disrupts epithelial barrier integrity, promotes microbial translocation, and drives the systemic release of inflammatory mediators. In parallel, gut dysbiosis alters transferable microbiota-derived signals, including SCFAs, tryptophan metabolites, and extracellular vesicles. These intestine-derived cues may influence the lungs through four interconnected pathways: systemic release of inflammatory mediators, immune-cell trafficking and cross-homing, circulating microbial metabolites and signals, and bone marrow hematopoiesis with peripheral immune reprogramming. Pulmonary consequences include (A) altered pulmonary immune microenvironment, (B) increased susceptibility to pulmonary inflammation, (C) subclinical pulmonary abnormalities, and (D) pulmonary function and radiologic abnormalities. In clinical practice, these disease-intrinsic pathways may overlap with pulmonary extraintestinal manifestations of IBD, infection, and drug-related pulmonary injury, thereby creating substantial diagnostic overlap and complicating the evaluation of respiratory abnormalities in patients with IBD. Created in BioRender. Mu, Z. (2026) https://BioRender.com/s7bes00.

In summary, IBD-related pulmonary injury is characterized by high insidiousness, phenotypic heterogeneity, and multi-pathway mechanistic coupling. Elucidating its pathogenesis will not only help uncover the inter-organ regulation mechanisms of the mucosal immune system but also provide a theoretical basis for early clinical screening and warning, precise classification, and targeted intervention. This article systematically integrates existing evidence and proposes verifiable research frameworks and future translational directions along four main lines: intestinal barrier, bidirectional regulation of the gut–lung axis, effects of microbiota metabolites on pulmonary immunity, and drug-related lung injury (5, 19, 20, 22). The hierarchical relationships among these mechanisms and their connections with overlaps in clinical diagnosis are summarized in **Figure 1**.

## 2 Intestinal barrier dysfunction as the initiating event

The skin, lungs, and gut together compose the three major barrier interfaces of the body with the external environment. Among these, the gut possesses the largest exposed surface area and is persistently subjected to a high antigenic load from the combined presence of commensal microbes, dietary components, and environmental antigens, making barrier homeostasis fundamentally important for systemic immune balance (17, 26). The onset and persistence of IBD are closely related to impairment of the intestinal mucosal barrier. In addition to its roles in nutrient absorption and digestion-associated transport, the intestinal epithelium acts as a crucial selective defense barrier, separating luminal contents from the immune cell network within the lamina propria, thereby limiting the abnormal translocation of microbes and their products, as well as excessive immune activation (26, 27). The intestinal epithelial barrier primarily consists of a continuous monolayer of specialized intestinal epithelial cells (IECs), with a highly organized paracellular pathway regulation system formed by intercellular junctional complexes. TJs are located in the apicolateral membrane region and constitute the rate-limiting structure for paracellular permeability (26, 28). TJs are not absolutely sealed bands but rather achieve selective permeability to water, electrolytes, and solutes through diverse combinations of transmembrane and scaffolding proteins. Their core components include the claudin family, occludin, junctional adhesion molecules (JAMs), as well as scaffolding proteins such as zonula occludens-1 (ZO-1)/zonula occludens-2 (ZO-2) that form a coupled network with the cytoskeleton (actin) (26, 28). Notably, the baseline permeability and regulatory capacity of TJs differs significantly across tissues (e.g., skin/bladder versus intestine/renal tubule); intestinal TJs physiologically permit a certain degree of paracellular flux to accommodate absorption and fluid transport, while remaining subject to highly specific regulation by immune signals, thus achieving a dynamic balance between absorption and defense (29).

In chronic inflammatory conditions, such as IBD, cytokine networks (particularly tumor necrosis factor, TNF) can regulate cytoskeletal tension and the localization/internalization of tight junction proteins, thereby affecting the pore pathway and leak pathway, respectively, leading to increased paracellular permeability and diminished barrier selectivity (26, 29, 30). This change is not merely a consequence of inflammation but may also promote immune activation. Barrier defects facilitate the translocation of luminal microbial products, including PAMPs and antigens, across the mucosa, thereby triggering sustained activation of immune cells within the lamina propria and the release of pro-inflammatory mediators. This process may establish a feed-forward amplification loop in which increased intestinal permeability, immune activation, and further barrier disruption reinforce one another (26, 31). Strong experimental evidence comes from the targeted manipulation of epithelial tight junctions: Su et al. demonstrated that inducing epithelial tight junction dysfunction alone can trigger immune activation and promote experimental colitis, thus supporting the view that “barrier defects can serve as pro-inflammatory drivers” (31). At the molecular level, in patients with active CD, upregulation of the pore-forming claudin-2, alterations of barrier-forming claudins, and disruption of TJ continuity are closely associated with barrier dysfunction (15). Therefore, barrier failure itself may not directly cause any specific pulmonary pathology; however, it provides an essential prerequisite for all subsequent, more specific gut–lung axis mechanisms.

**Figure 2:**
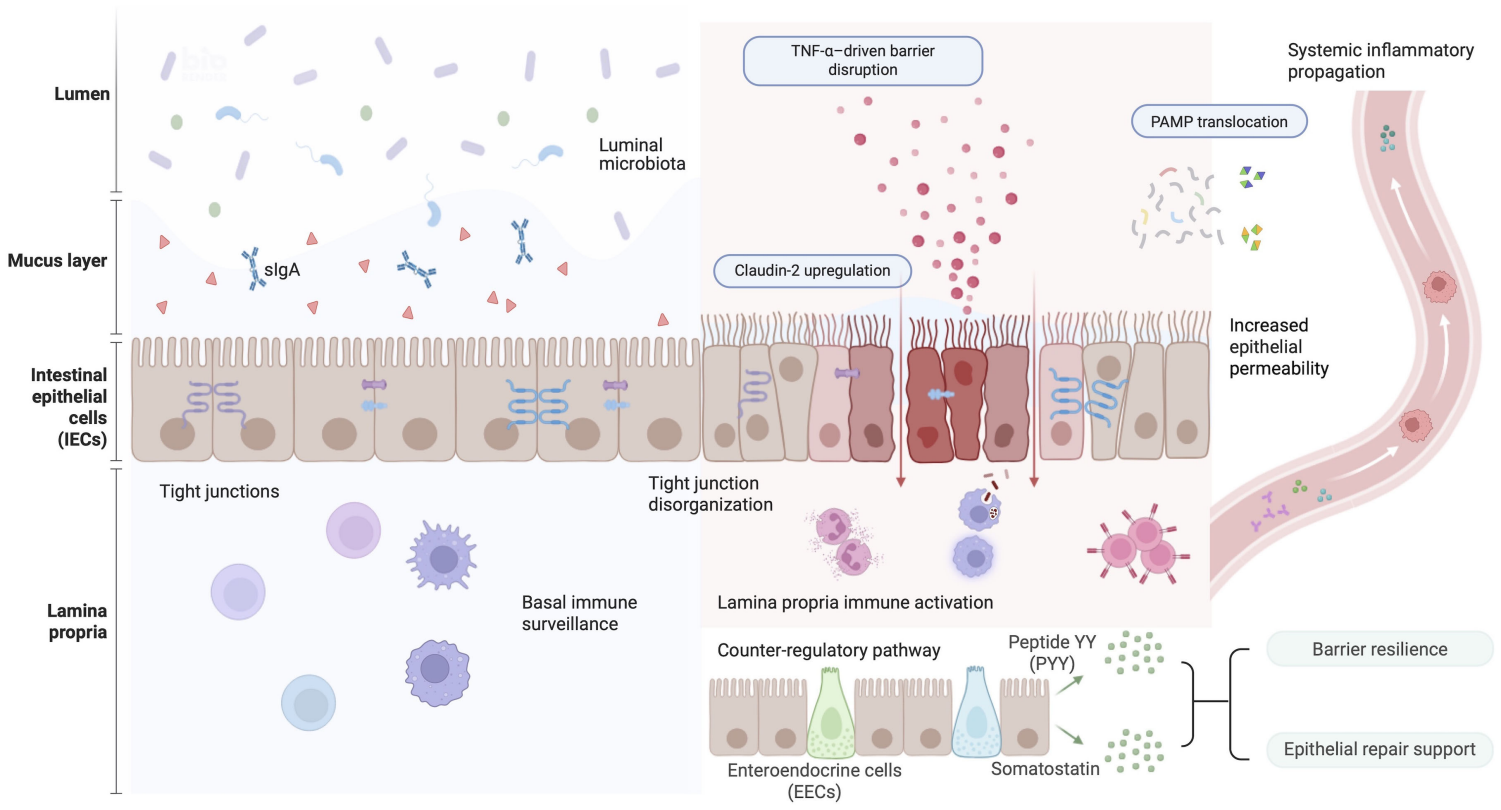
Intestinal barrier failure as an initiating event linking gut inflammation to systemic and pulmonary injury. Under homeostatic conditions, the intestinal barrier is maintained by the mucus layer, secretory IgA, intestinal epithelial cells (IECs), intact tight junctions, and basal immune surveillance, thereby restricting luminal microbiota to the intestinal lumen. In IBD, TNF-α-driven barrier injury and Claudin-2 upregulation are associated with tight junction disorganization and increased epithelial permeability, facilitating the translocation of PAMPs and other luminal microbial products into the lamina propria. This promotes local immune activation, amplifies intestinal inflammation, and may contribute to systemic inflammatory propagation. In parallel, enteroendocrine cells (EECs) and their peptide mediators, including peptide YY (PYY) and somatostatin, may provide counter-regulatory support for barrier resilience and epithelial repair. Together, these processes support intestinal barrier failure as a mechanistically central event that enables inflammatory and microbial signals to extend beyond the gut, with potential downstream relevance to remote pulmonary injury in IBD. Created in BioRender. Mu, Z. (2026) https://BioRender.com/nfo4tox.

Traditionally, barrier research has focused largely on goblet/Paneth cells and classical tight-junction molecules. However, recent evidence suggests that enteroendocrine cells (EECs) may be key modulators that enhance barrier resilience under inflammatory conditions. EECs constitute approximately 1% of IECs and, functioning as sensor cells, respond to nutrient cues and microbial metabolites by secreting various peptide hormones, thereby mediating immunoendocrine regulation at the epithelium–immune interface (32). Using human intestinal epithelial models, Nwako et al. showed that the loss of EECs significantly decreases transepithelial electrical resistance (TEER) and increases permeability. Supplementation with EEC secretory products—peptide YY (PYY) or somatostatin and its analogues —can improve barrier function under homeostatic conditions and in TNF-induced barrier injury (33–35). More importantly, the study indicated that barrier improvement does not greatly depend on a marked increase in the abundance of TJ proteins, such as ZO-1 or occludin, but may involve unknown mechanisms related to TJ ultrastructure, membrane protein recycling/endocytosis dynamics, or complex stability (33). Inconsistent *in vivo* studies by Kuo et al. also suggest that the deficiency of certain TJ scaffold proteins (e.g., ZO-1) does not necessarily cause catastrophic barrier collapse at baseline but is crucial for mucosal repair and post-inflammatory barrier reconstruction, underscoring that the “protein abundance–barrier function” relationship is not linear (36). Therefore, incorporating the EEC–peptide hormone axis into the mechanistic framework of IBD barrier injury and repair not only complements the traditional tight-junction model but also helps to explain how reduced barrier resilience is associated with TNF-driven increases in permeability, PAMP translocation, lamina propria immune activation, and subsequent systemic dissemination (**Figure 2**).

From a clinical and translational standpoint, the barrier-centered model can be partially operationalized through biomarkers of intestinal permeability and microbial translocation. Functional permeability tests, particularly the urinary lactulose/mannitol ratio, provide a direct assessment of altered intestinal barrier function (37, 38). In parallel, circulating or fecal biomarkers such as zonulin or zonulin-related proteins, intestinal fatty acid-binding protein (I-FABP/FABP2), lipopolysaccharide(LPS)/endotoxin, lipopolysaccharide-binding protein (LBP), soluble CD14 (sCD14), endotoxin-core antibodies (EndoCAb), bacterial DNA, and microbial cell-free DNA (mcfDNA) may capture distinct but overlapping aspects of epithelial injury, paracellular permeability, and systemic exposure to microbial products (38–41). These biomarkers should be interpreted cautiously rather than interchangeably. For example, zonulin-related assays may lack specificity for pre-haptoglobin-2/zonulin, whereas I-FABP more directly reflects enterocyte injury rather than paracellular permeability alone (38–40). Human IBD studies have reported increased circulating endotoxin, LBP, and sCD14 in association with disease activity and inflammatory cytokine responses (42).

Beyond cross-sectional inflammatory activity, serum LBP and sCD14 in Crohn’s disease have also been linked to subsequent clinical flare risk (43). More recent data further suggest that serum LBP may reflect endoscopic activity and correlate with C-reactive protein (CRP) and fecal calprotectin (44). Consistently, bacterial DNA concentrations are increased in blood and tissue samples from patients with IBD, particularly during active disease, and circulating bacterial DNA has been identified as an independent risk factor for short-term relapse and hospitalization in Crohn’s disease (45, 46). Together, these findings support the concept that intestinal barrier failure may permit microbial products and microbial nucleic acids to enter the systemic circulation, engage LPS–LBP–CD14/TLR4 signaling and innate pathways responsive to bacterial DNA, and amplify systemic inflammatory responses. However, direct evidence linking permeability or microbial-translocation biomarkers to objective pulmonary endpoints in IBD remains limited (42, 43). Future translational studies should therefore integrate standardized permeability testing, quantitative LPS/LBP/sCD14 and bacterial DNA/mcfDNA assays, inflammatory mediators, and pulmonary outcomes, including spirometry, DLCO, HRCT, sputum or bronchoalveolar lavage (BAL) inflammatory mediators, and longitudinal treatment response (42–44). Because blood and lower-airway samples are low-biomass microbial matrices, bacterial DNA- or mcfDNA-based studies should include rigorous negative controls, quantitative microbial-load assessment, contamination-aware sequencing pipelines, and validation in independent longitudinal cohorts (47).

## 3 Microbiota dysbiosis and transferable inflammatory signals

The human gut microbiota is a highly complex and dynamic cross-kingdom symbiotic ecosystem that includes fungi (mycobiota), viruses (virome), archaea, and some commensal protozoa in addition to bacteria. Its composition and function are collectively shaped by developmental stage, dietary patterns, drug exposure, geographic environment, and host genetic background, and it participates in the regulation of local and systemic host homeostasis through mechanisms such as nutritional metabolism, barrier maintenance, and immune education (45–47). In IBD research, merely describing increases or decreases in species is insufficient to account for disease heterogeneity; a more translational perspective is to view the microecosystem as a modifiable functional organ, emphasizing that its metabolic output, immune regulation, and tissue barrier interactions together determine the inflammatory phenotype (45, 46).

Dysbiosis is one of the core features of IBD. Early molecular phylogenetic studies and subsequent large cohort evidence have consistently indicated that patients with IBD often present with reduced microbial diversity, a decrease in certain strictly anaerobic commensals, and enrichment of facultative anaerobes, such as Enterobacteriaceae (48–50). In newly diagnosed, treatment-naïve patients with CD, this pattern of “reduced protective anaerobes plus enriched Enterobacteriaceae” is more pronounced and correlates with disease location and activity (49, 50). Recent research has further confirmed that *Faecalibacterium prausnitzii*, an important anti-inflammatory commensal bacterium, is decreased in IBD and can directly induce monocytes to produce IL-10 and remodel immunometabolism without eliciting significant pro-inflammatory responses, supporting its protective role in the regulation of intestinal inflammation (51).

It should be emphasized that microbial ecological abnormalities in IBD have a distinct temporal dimension and volatility. Longitudinal studies have shown that the microbiota of patients with IBD is less stable over time than that of healthy individuals and is dynamically coupled with disease relapse and interventions (50). At the functional level, integrated metagenomic and transcriptomic research further suggests that IBD is not only about microbiota change but also that the functional output of the microbiota has changed, including systematic shifts in metabolic pathways and immune-related molecular patterns (46, 52). This concept of functional reprogramming is particularly critical for the subsequent discussion of the gut–lung axis, as cross-organ effects often depend more on transferable microbial components and metabolites than on the mere presence or absence of individual species (53).

Probiotics are defined by the International Scientific Association for Probiotics and Prebiotics (ISAPP) consensus as “live microorganisms that, when administered in adequate amounts, confer a health benefit on the host,” but the effects of different strains vary significantly and cannot be simply extrapolated at the genus or phylum level (54). Mechanistically, most probiotic/commensal bacteria can enhance the mucus layer, promote the production of antimicrobial peptides and secretory IgA (sIgA), modulate tight junction-related signaling, and inhibit pathogenic bacterial adhesion, thereby improving epithelial barrier function and buffering amplification of inflammation (55, 56). Immunologically, Lactobacillus and similar bacteria can interact with epithelial cells and mucosal immune cells, modulate Toll-like receptor (TLR) signaling and the cytokine profile, and thus adjust the threshold of innate and adaptive immune responses (56).

It is noteworthy that microecological interventions and distal organ immunity are not isolated from each other. Within the framework of the CMIS/gut–lung axis, specific strains can exhibit cross-organ protective effects in respiratory tract infection and secondary bacterial challenge models. For example, Clua et al. demonstrated in infant mouse models that nasal pretreatment with non-viable *Lactobacillus rhamnosus* CRL1505 or its peptidoglycan can enhance resistance to respiratory syncytial virus infection and secondary Streptococcus pneumoniae infection, indicating that alveolar macrophages play a critical role in this protective effect (57). Collectively, these findings provide experimental support for a plausible pathway in which microecological signals calibrate mucosal immune responses and thereby alter the threshold for pulmonary inflammation. This mechanism may help explain altered pulmonary susceptibility in patients with IBD (53, 57).

IBD-associated dysbiosis is reflected not only in reduction of beneficial bacteria but also in the expansion and pro-inflammatory synergy of specific pathobionts in an inflammatory milieu. Adherent-invasive *Escherichia coli* (AIEC) is considered to be closely associated with ileal-type CD, capable of invading epithelial cells and surviving and replicating within macrophages, thereby inducing the release of pro-inflammatory factors such as TNF-α and sustaining inflammation (58). At the level of host susceptibility, epithelial carcinoembryonic antigen-related cell adhesion molecule 6 (CEACAM6) can serve as an adhesion receptor for AIEC and is aberrantly expressed in the ileal epithelium of CD; relevant transgenic models have confirmed that AIEC can achieve persistent colonization and induce pronounced intestinal inflammation via fimbriae–CEACAM interactions (59, 60). Additionally, inflammation alters the intestinal ecological niche (including the redox environment and available electron acceptors), conferring a competitive advantage to facultative anaerobes (such as Enterobacteriaceae) under inflammatory conditions. A series of studies in Science demonstrated that inflammation-associated metabolic reprogramming promotes the expansion of Enterobacteriaceae (e.g., host-derived nitrate facilitating *E. coli* growth; PPARγ-related pathways affecting niche competition), providing a robust mechanistic foundation for a positive feedback loop of inflammation–dysbiosis–further inflammation (61, 62). In the context of IBD-associated lung injury, a key extrapolation of such changes is that Enterobacteriaceae expansion frequently coincides with an increased endotoxin burden and mucosal translocation risk, offering a microbiological starting point for the subsequent hypothesized pathway by which gut-derived inflammatory signals affect pulmonary immune thresholds (53).

The role of mycobiota in IBD has received increased attention in recent years. A study in Science revealed that Dectin-1 deficiency, a key host–fungal recognition receptor, increases susceptibility to colitis, supporting a decisive role for the fungus–host receptor axis in the severity of mucosal inflammation (63). Review evidence has also highlighted that cross-kingdom interactions between fungi and bacteria are important variables that shape mucosal immune homeostasis and inflammatory phenotypes (64). Taking *Candida albicans* as an example, its morphological transitions and virulence factors can directly damage the epithelium and amplify inflammation. Nature identified candidalysin as a critical mucosal cytotoxin that drives epithelial injury and inflammatory responses; subsequent work further showed that it can activate the epidermal growth factor receptor (EGFR)–mitogen-activated protein kinase (MAPK) signaling pathway in epithelial cells to amplify host responses (65, 66). Meanwhile, *C. albicans* adhesins and invasins, such as Als3, can bind to host cadherins and induce endocytosis, providing a molecular basis for transepithelial invasion (67). More direct support for “cross-kingdom interactions–distal pulmonary inflammation” comes from recent mechanistic studies: Wang et al. showed that antifungal-induced gut fungal dysbiosis promotes Enterobacteriaceae overgrowth and subsequent translocation of *E. coli* to the lung during infection. This response was accompanied by the accumulation of distinct pulmonary macrophage populations and exacerbated lung inflammation in a TLR4-dependent manner. Macrophage depletion or TLR4 deficiency abolished this effect, supporting a mechanistic link between fungal dysbiosis, bacterial translocation, and amplified pulmonary inflammation (68). This study has important implications for IBD-associated lung injury: under immunosuppressive therapy or infectious stress, cross-kingdom dysbiosis and translocation mechanisms may serve as major upstream drivers of pulmonary inflammation susceptibility (53, 68).

Bacteria can release outer membrane vesicles (OMVs) and other extracellular vesicles/membrane vesicles as nanoscale lipid bilayer carriers to transport proteins, lipids, nucleic acids, and metabolites, thereby mediating intercellular signaling and modulating host immune responses (69). A Nat Rev Immunol review pointed out that bacterial vesicles can exert bidirectional regulatory effects in host–microbe interactions, capable of delivering both pro-inflammatory signals and inducing immune tolerance and barrier protection; thus, they constitute an important direction for understanding the inflammatory network of IBD and developing novel intervention strategies (70). In the translational context, the consensus definition of “postbiotics” by ISAPP emphasizes that inactivated microbial preparations and their components can also confer health benefits, suggesting that controlled signal components—including bacterial EVs—may outperform live biotic interventions in terms of safety and reproducibility (71).

In addition to bacteria and fungi, commensal protozoa may also participate in shaping baseline mucosal immunity. A cell study showed that the commensal protozoan *Tritrichomonas musculis* can activate epithelial inflammasomes and induce interleukin-18 (IL-18) release, thereby promoting dendritic cell-driven T helper 1/T helper 17 (Th1/Th17) immunity and enhancing defense against mucosal bacterial infection; however, it may also exacerbate T cell–mediated colitis and tumor susceptibility, displaying a protective yet costly immune ecological profile (72). Within the framework of IBD-associated lung injury, whether baseline mucosal immune remodeling factors influence pulmonary inflammatory thresholds via systemic immune reprogramming remains to be validated through longitudinal cohort studies and mechanistic experiments (53, 72).

Overall, the key to IBD-associated dysbiosis lies not merely in altered species composition, but more importantly in systematic reprogramming of functional outputs and transmittable signals, including the expansion of pathobionts, disruptions in cross-kingdom interactions, heightened risk of translocation, and the involvement of vesicular/postbiotic signals in immune regulation (46, 52, 68, 70, 71). Among these functional outputs, metabolites represented by SCFAs stand out as pivotal nodes for further discussion, due to their direct coupling of diet–microbiota function, well-defined receptors and epigenetic pathways, and substantial preclinical and translational evidence regarding the gut–lung axis (52, 53, 73).

## 4 Microbiota–metabolite–immune remodeling along the gut–lung axis

An increasing body of clinical and experimental research supports that the gut and lung are not independent mucosal organs, but rather form a continuous bidirectional crosstalk network via the gut–lung axis. At the heart of this axis, the microbiota and their metabolites, circulating inflammatory mediators, and immune cell trafficking collectively shape the distal mucosal immune threshold and tissue homeostasis (53, 74). Within this framework, signals originating from the gut impact pulmonary immunity mainly through the following mechanisms (1): microbial metabolites (such as SCFAs) that, after entering circulation, act directly on the lung epithelium or immune cells (2); gut inflammation, which leads to increased barrier permeability and microbial translocation, driving systemic inflammation and altering the baseline of pulmonary immunity; and (3) remote programming of bone marrow hematopoietic and peripheral immune cell lineages under the influence of microbiota-derived signals, thereby modifying the intensity and type of pulmonary inflammatory responses (18, 53, 74). Therefore, IBD-associated dysbiosis may reduce the capacity of pulmonary mucosal defense via gut-derived signals and may amplify lung inflammatory injury under stress conditions such as infection, drug exposure, or hypoxia, constituting a critical upstream context for IBD-related lung injury (53, 74).

Gut microbiota-derived anaerobic fermentation of dietary fibers, such as resistant starch, pectin, and cellulose, generates SCFAs. This metabolite class generally comprises fatty acids with chain lengths of six or fewer carbon atoms, including acetate (C2), propionate (C3),butyrate (C4), and small amounts of valerate. They are present in the highest concentrations in the colon, with part being absorbed by the epithelium and entering the portal vein and systemic circulation, acting as transportable metabolic-immune signaling molecules to influence distal organs (18, 75). From an ecological perspective, SCFA production depends on a complex cross-feeding network, in which intermediates, such as lactate and acetate, can provide substrates and an acidic ecological niche for butyrate producers; metabolic cooperation among different microorganisms (bacteria/archaea, etc.) determines the quantity and proportion of SCFA profiles (18). Conversely, SCFAs indirectly shape the structure and stability of microbial communities by altering luminal pH, inhibiting the virulence of certain pathogens, and modulating host immune responses (18, 75).The canonical receptors for SCFAs include free fatty acid receptor 2 (FFAR2), free fatty acid receptor 3 (FFAR3), and hydroxycarboxylic acid receptor 2 (HCAR2). These receptors are expressed on intestinal epithelial cells, enteroendocrine cells, and various immune cell types, enabling SCFAs to rapidly transduce signals along the epithelial-immune axis and reset the inflammatory response threshold (75–77). Animal models suggest that FFAR2-mediated SCFA signaling is closely linked to inflammation resolution: Maslowski et al. reported in Nature that FFAR2-deficient mice exhibit exacerbated or more persistent inflammatory responses in multiple inflammation models, supporting the idea that SCFA–FFAR2 represents an important bridge linking diet, microbial metabolism, and immune homeostasis (76).Trompette et al. demonstrated that a high-fiber diet increases circulating SCFAs and alleviates allergic airway inflammation, with key effects attributable to FFAR3 -dependent alterations in bone marrow hematopoiesis and dendritic cell lineages, indicating that SCFAs act not only locally in the gut but can also reshape the pulmonary immune landscape via a bone marrow–lung axis (19).Propionate and butyrate are widely regarded as HDAC inhibitors that influence gene transcription by increasing histone acetylation levels, and thus play critical roles in Treg differentiation, anti-inflammatory cytokine expression, and the regulation of inflammatory signaling pathways (18, 75). In experimental studies related to lung inflammation, butyrate, as an HDAC inhibitor, can alleviate LPS-induced acute lung injury and suppress nuclear factor-κB (NF-κB) -mediated proinflammatory amplification, providing a verifiable mechanistic basis for the link between SCFAs–epigenetics–pulmonary inflammation (78). At the level of antiviral defense in the respiratory tract, Antunes et al. reported in Nat Commun that acetate produced by a high-fiber diet/gut microbiota can, via FFAR2, enhance lung epithelial interferon-beta (IFN-β)–related antiviral gene expression, thereby reducing the severity of respiratory syncytial virus (RSV) infection, suggesting that SCFAs may act as gut-derived baseline regulators of antiviral responses (79).

In addition to immune modulation, SCFAs participate directly in the maintenance of barrier homeostasis. Kelly et al. proposed in Cell Host & Microbe the mechanism of “butyrate metabolism–oxygen consumption–hypoxia-inducible factor stabilization,” in which microbiota-derived butyrate promotes colon epithelial oxygen consumption and stabilizes hypoxia-inducible factor (HIF), thereby activating barrier-protective transcriptional programs. When HIF is deficient, the barrier-protective effect of butyrate is significantly diminished, indicating that HIF is a critical node for butyrate-mediated barrier resilience (80). At the level of tight junctions, Zheng et al. reported that physiological concentrations of butyrate can suppress Claudin-2 expression, which promotes permeability, and enhance epithelial barrier function via an IL-10 receptor α (IL-10RA)-mediated mechanism, providing more specific molecular evidence for SCFAs–immune–epithelial coupling (81). For IBD, this implies that reduced levels of SCFAs may not only reflect insufficient functional output of the microbiome, but, more importantly, could compromise barrier integrity and anti-inflammatory programs, indirectly increasing the risk of microbial translocation and systemic inflammatory spillover, thereby fostering susceptibility to and amplification of pulmonary inflammation and injury (18, 53, 80).

Although SCFAs form the main axis of this section, microbially derived metabolites from tryptophan should also be considered an important secondary branch. Microbial tryptophan metabolites can act as endogenous ligands for AhR, influencing antigen presentation, T-cell differentiation, and innate lymphoid cell activation, thereby regulating mucosal immune homeostasis and barrier repair (82, 83). Classic studies have shown that microbial tryptophan metabolites can balance mucosal reactivity and enhance barrier-associated defense programs by activating the AhR–IL-22 axis (82). Further research has indicated that metabolite production is strain-specific; for example, indole-3-lactic acid (ILA) can improve experimental colitis phenotypes via AhR-related pathways, highlighting the importance of the strain–metabolite–receptor triad in mechanistic inference and precision intervention (84). Meanwhile, another highly consistent functional alteration in IBD is the abnormal profile of key metabolites, such as SCFAs, which are believed to shape mucosal and systemic immune baselines via receptor signaling and epigenetic regulation, and are closely linked to distal effects along the gut–lung axis (52, 53).

Beyond SCFAs and microbial tryptophan-derived indole metabolites, additional microbiota-modulated metabolic pathways may further expand the gut–lung axis from a model centered on SCFAs to a broader multi-metabolite network. Among these pathways, bile acid remodeling represents a major host–microbiota co-metabolic process. Primary bile acids synthesized by the host liver undergo microbial deconjugation, dehydroxylation, and other biotransformations in the intestine, generating secondary bile acids with potent signaling properties (85). These metabolites may regulate epithelial barrier integrity, antimicrobial responses, innate immune tone, and adaptive immune polarization through the farnesoid X receptor (FXR), G protein-coupled bile acid receptor 1 (TGR5/GPBAR1), and other bile acid-sensitive signaling pathways (86–88). In particular, lithocholic acid-derived metabolites have been shown to directly modulate T cell differentiation, with 3-oxoLCA suppressing Th17 cell differentiation and isoalloLCA promoting regulatory Treg differentiation, supporting a mechanistic link between microbial bile acid metabolism and the Th17/Treg balance (86). Therefore, impaired secondary bile acid generation in IBD may plausibly contribute to mucosal immune dysregulation and systemic inflammatory propagation, although direct evidence connecting this pathway to IBD-associated pulmonary injury remains limited.

Microbiota-associated lipid mediators provide an additional layer of immune regulation. Bacterial sphingolipids, particularly those derived from Bacteroides species, have been implicated in intestinal homeostasis; however, their effects appear to be highly context-dependent (89). For example, Bacteroides fragilis-derived sphingolipids were recently reported to exacerbate experimental colitis by restricting epithelial IL-18 expression and suppressing IL-22 production by type 3 innate lymphoid cells (ILC3s), thereby impairing mucosal repair responses (90). Succinate should also be interpreted as a context-dependent immunometabolic signal rather than a purely microbial product, because it can arise from both host mitochondrial metabolism and microbial fermentation. Under dysbiotic conditions, an imbalance between succinate-producing and succinate-consuming bacteria may lead to the accumulation of succinate. Experimental evidence has linked gut microbiota-derived succinate to acute lung injury through succinate receptor 1 (SUCNR1)-associated alveolar macrophage polarization, epithelial apoptosis, and pulmonary inflammation (91). More recently, studies in ulcerative colitis identified microbiota-derived succinate as a driver of colitogenic helper T cell responses through the succinate–SUCNR1–IL-9 axis, while blockade of SUCNR1 signaling, neutralization of IL-9, or restoration of succinate-consuming bacteria attenuated experimental inflammation (92). In addition, succinate has been shown to promote intestinal inflammation by destabilizing forkhead box P3 (FOXP3) and impairing Treg function, further supporting its pathogenic potential in IBD (93).

The TMA/TMAO pathway represents a microbiota-dependent host–microbial co-metabolic axis. TMA is generated by gut microbial metabolism of dietary substrates such as choline and carnitine, whereas TMAO is produced primarily after hepatic oxidation of TMA by flavin-containing monooxygenases (94). At present, direct evidence linking TMAO to IBD-associated pulmonary injury is scarce and heterogeneous. Some ulcerative colitis cohorts have even reported reduced circulating TMAO levels compared with healthy controls, highlighting disease-stage, dietary, microbial, and host-metabolic variability (95). Nevertheless, the TMA/TMAO axis remains relevant to gut–lung research because TMAO has been implicated in endothelial dysfunction, oxidative stress, NLRP3 inflammasome activation, vascular injury, and pro-fibrotic signaling in other inflammatory and cardiopulmonary contexts (94, 96). Taken together, these metabolites should be regarded as emerging or supportive mechanisms within the gut–lung axis rather than established central drivers of IBD-associated pulmonary injury. Their inclusion helps broaden the mechanistic framework, but their causal relevance requires validation in studies integrating intestinal metabolomics with pulmonary endpoints, including lung function, HRCT, bronchoalveolar or sputum immune profiling, and longitudinal treatment response.

The common mucosal immune system provides a trafficking-based framework through which intestinal inflammation may influence distant mucosal organs, including the lung. During intestinal priming, gut-associated dendritic cells, particularly under retinoic acid-dependent cues, can imprint activated lymphocytes with gut-tropic receptors, most notably α4β7 integrin and CCR9 (97). α4β7 binds mucosal addressin cell adhesion molecule-1 (MAdCAM-1) on intestinal endothelium, whereas CCR9 responds to CCL25; together, these pathways mediate mucosal immune surveillance and constitute a central therapeutic axis in IBD, as illustrated by α4β7-targeted therapy (98, 99). However, under persistent inflammatory conditions, tissue-specific trafficking programs may become more permissive rather than strictly organ-restricted. The best human precedent comes from IBD-associated hepatobiliary disease, where aberrant hepatic endothelial expression of MAdCAM-1 and CCL25 supports recruitment of α4β7^+^CCR9^+^ gut-homing lymphocytes to the liver (100, 101). By analogy, a similar ectopic mucosal-homing program may contribute to pulmonary involvement in IBD, although direct evidence from human BAL fluid or lung tissue remains limited. Experimental colitis studies support this biological plausibility: chronic UC-like inflammation in rats increased pulmonary Madcam1 mRNA, and a recent dextran sulfate sodium (DSS) mouse model showed intestinal barrier disruption accompanied by microbial translocation, lung inflammation, and accumulation of α4β7^+^ and CCR9-expressing cluster of differentiation 4-positive T cells (CCR9^+^CD4^+^ T cells) in the lung (102, 103).

Chemokine axes add another layer of specificity. The CCR6–CCL20 pathway is closely linked to recruitment of IL-17-secreting innate lymphoid cells and Th17 cells; intestinal epithelial CCL20 and CCR6 are increased during active ulcerative colitis and Crohn’s disease, and recent clinical data further suggest systemic CCL20 elevation in IBD (104–106). CXCR3 and its interferon-inducible ligands CXCL9, CXCL10, and CXCL11 may further recruit Th1-polarized and Th17/Th1-like effector cells to inflamed mucosal tissues (104). This is relevant because IL-17-family responses can promote neutrophil recruitment and mucosal antimicrobial programs but, when dysregulated, may also amplify tissue inflammation (107). Recent single-cell and spatial profiling of IBD gut biopsies has identified interferon-response programs that co-localize with T-cell aggregates and epithelial damage, supporting the existence of organized inflammatory trafficking niches in the intestinal mucosa (108). Whether analogous immune niches exist in IBD-affected lungs remains unknown.

Once effector T cells reach the lung, pulmonary microenvironmental cues may promote retention and local immunological memory. Lung tissue-resident memory T cells (TRM cells) are commonly characterized by the expression of cluster of differentiation 69 (CD69), with integrin αE (CD103) often marking epithelial-associated subsets; their persistence can confer barrier protection but may also sustain recurrent local inflammation when antigenic or cytokine stimulation persists (109). Therefore, in IBD-associated pulmonary injury, aberrant immune-cell homing should be conceptualized not as a proven linear gut-to-lung migration pathway in humans, but as a testable model in which intestinal priming, systemic inflammatory chemokines, ectopic endothelial addressins, and local tissue-resident memory formation converge to maintain pulmonary immune dysregulation.

**Figure 3:**
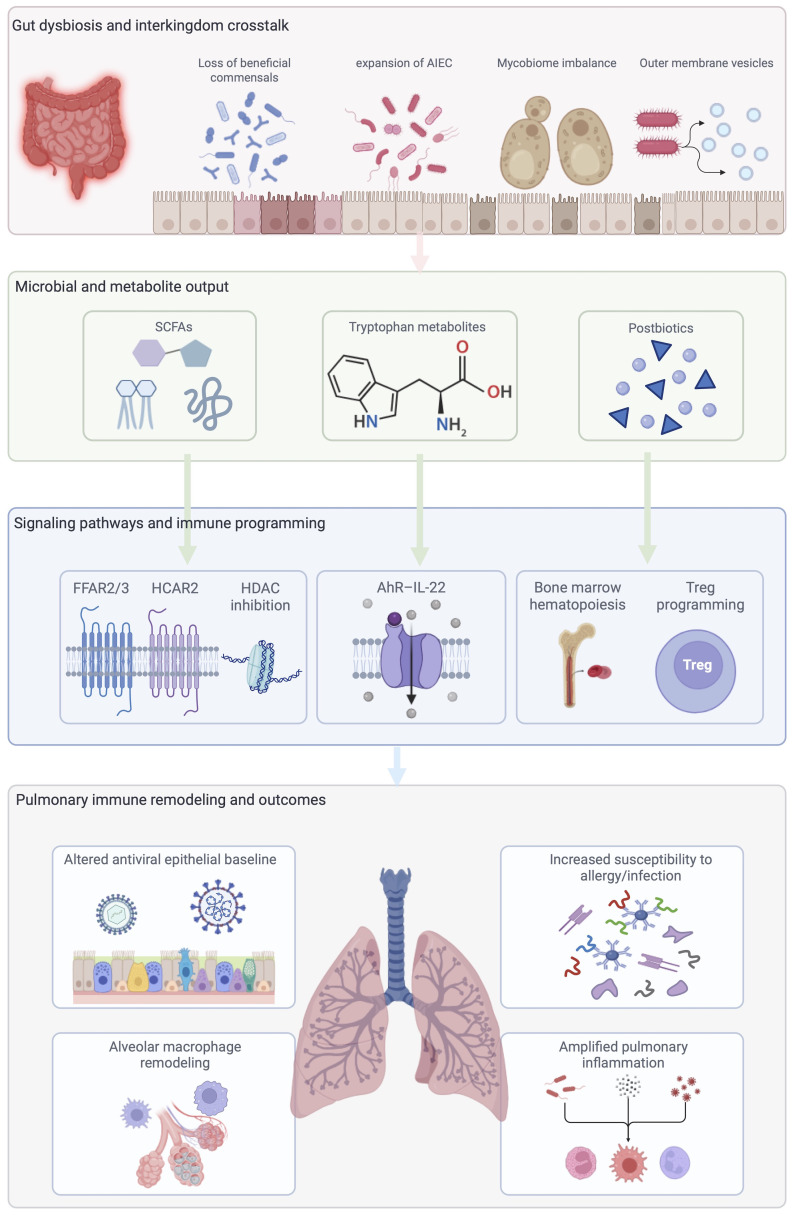
Microbiota-derived signals and metabolite-centered immune remodeling along the gut–lung axis in IBD. Gut dysbiosis in IBD, characterized by the loss of beneficial commensals, expansion of AIEC, mycobiome imbalance, and outer membrane vesicle release, reshapes the output of microbiota-derived mediators, including short-chain fatty acids, tryptophan metabolites, and postbiotics. These signals engage receptor-mediated and epigenetic pathways, including FFAR2/3, HCAR2, HDAC inhibition, and AhR–IL-22 signaling, and converge on immune-programming processes such as bone marrow hematopoiesis and Treg programming. Through these interconnected mechanisms, intestinal microbial perturbation may remodel pulmonary immunity, altering the antiviral epithelial baseline, alveolar macrophage phenotype, susceptibility to allergy or infection, and the amplification of pulmonary inflammation. AIEC, adherent-invasive *Escherichia coli*; AhR, aryl hydrocarbon receptor; FFAR, free fatty acid receptor; HCAR2, hydroxycarboxylic acid receptor 2; HDAC, histone deacetylase; IBD, inflammatory bowel disease; OMVs, outer membrane vesicles; Postbiotics, non-viable microbial cells, cell components, or microbial-derived products with potential biological activity; SCFAs, short-chain fatty acids; Treg, regulatory T cell. Created in BioRender. Mu, Z. (2026) https://BioRender.com/2tyry62.

The gut–lung axis is not a unidirectional pathway. The pulmonary microbiota itself is a low-biomass but highly dynamic ecosystem, with its community structure collectively determined by three ecological factors: microbial immigration, elimination, and relative reproduction rates (110). In healthy states, community membership is primarily governed by a balance between immigration and elimination; in disease states, altered local growth conditions may promote the abnormal expansion of specific bacterial populations (111–113). In terms of origin, multiple studies have suggested that the healthy pulmonary bacterial community is more similar to the oropharyngeal microbiota, with subclinical microaspiration and mucociliary clearance shaping its homeostasis (113–116). When mucus secretion increases, ciliary function is impaired, or local inflammatory cell infiltration changes, pulmonary niches may shift toward conditions more favorable for the expansion of particular bacterial groups (117). More crucially, respiratory viral infections can disrupt gut microbiota and immunity via immunometabolic pathways in a reverse manner. Wang et al. demonstrated that respiratory influenza infection can induce intestinal immune injury via the migration of lung-derived CCR9^+^CD4^+^ T cells to the small intestine. These cells promoted interferon-γ (IFN-γ)- mediated alterations in the gut microbiota, leading to Th17-associated intestinal inflammation and mucosal damage. This finding provides experimental evidence that pulmonary inflammation can reshape intestinal ecology and mucosal immune homeostasis through immune-cell trafficking and cytokine-dependent signaling (118). Deriu et al. further showed that influenza-associated type I interferon signaling alters the gut microbiota and increases susceptibility to secondary intestinal *Salmonella* infection. These findings highlight the systemic consequences of pulmonary antiviral responses, suggesting that respiratory infection can remodel intestinal niches and thereby compromise host resistance to secondary enteric infection (119). In addition, review evidence suggests that appetite loss (leading to reduced fiber intake), changes in systemic inflammatory mediators, and altered gut metabolic environment following respiratory viral infection can cause gut dysbiosis and reduce the production of metabolites, such as SCFAs. This process may subsequently impair pulmonary host defense against secondary bacterial infection, thereby establishing a bidirectional lung–gut–lung axis of immune and microbial crosstalk (74). Unlike intestinal dysbiosis, direct human evidence specifically demonstrating IBD-associated lower-airway microbiome alterations remains limited and has not yet been sufficiently validated in well-controlled studies (20, 120). Overall, these lines of evidence support understanding IBD-associated dysbiosis as a functional immune remodeling process: it can influence the baseline antiviral state of the pulmonary epithelium, the status of alveolar macrophages, susceptibility to infection/allergy, and amplification of inflammation via SCFAs, tryptophan metabolites, postbiotics/OMVs, and bone marrow–lung immune programming (**Figure 3**). To provide an integrated overview of the proposed gut–lung axis pathways and their translational relevance, key mechanisms, supporting evidence, and remaining gaps are summarized in **Table 1**.

**Table 1:** Proposed gut-lung axis mechanisms linking intestinal inflammation to pulmonary immune remodeling in IBD.

Proposed mechanism	Representative supporting evidence	Translational implications and remaining gaps
Barrier dysfunction and microbial translocation	IBD-associated permeability can permit gut-derived microbial products, including LPS and bacterial DNA, to enter circulation and promote systemic innate immune activation. Experimental colitis models further link barrier disruption with lung inflammation and microbial translocation.	Combine gut permeability and microbial-translocation biomarkers with pulmonary endpoints such as spirometry, DLCO, HRCT, sputum/BAL inflammation, and longitudinal treatment response.
SCFA-mediated metabolic-immune regulation	Microbiota-derived acetate, propionate, and butyrate regulate epithelial and immune responses through FFAR2, FFAR3, HCAR2, and HDAC-dependent pathways. High-fiber/SCFA signaling can reshape bone marrow hematopoiesis, antiviral epithelial tone, and airway inflammation (19, 76, 79).	SCFA profiling may help connect diet, dysbiosis, and pulmonary immune status. IBD-specific studies should test whether SCFA restoration improves lung outcomes.
Tryptophan-derived indole-AhR signaling	Microbial tryptophan metabolites activate AhR-related pathways, including AhR-IL-22 signaling, thereby supporting mucosal immune balance and barrier repair. These effects are strain- and metabolite-specific (82, 121).	Intestinal metabolomics could identify patients with impaired barrier-repair programs. Direct evidence linking this pathway to IBD-associated pulmonary injury remains limited.
Additional microbiota-modulated metabolites	Bile acid remodeling, bacterial sphingolipids, succinate, and the TMA/TMAO axis can influence Th17/Treg balance, epithelial repair, inflammasome activation, macrophage polarization, and vascular or fibrotic signaling in context-dependent ways (86, 87, 89, 90, 96).	These pathways should be presented as emerging or supportive mechanisms. Their causal relevance requires paired intestinal metabolomics and pulmonary phenotyping.
Immune-cell trafficking and inflammatory niches	The common mucosal immune system supports gut-primed lymphocyte trafficking through α4β7-MAdCAM-1 and CCR9-CCL25 pathways. Experimental colitis studies suggest pulmonary accumulation of gut-homing T-cell populations, while CCR6-CCL20 and CXCR3 ligands may organize inflammatory recruitment (97, 103–105).	Human BAL, lung tissue, or spatial profiling studies are needed to determine whether gut-imprinted trafficking programs and TRM-like retention sustain pulmonary inflammation in IBD.
Pulmonary dysbiosis and bidirectional lung-gut feedback	The lung microbiota is a low-biomass ecosystem shaped by immigration, elimination, and local growth. Respiratory viral infection can alter gut microbiota and intestinal immunity through cytokine- and immune-cell-dependent mechanisms (74, 118, 119, 122).	Future studies should evaluate gut and airway microbiomes together, rather than treating lung involvement as a purely downstream event.

AhR, aryl hydrocarbon receptor; BAL, bronchoalveolar lavage; CCL20, C-C motif chemokine ligand 20; CCL25, C-C motif chemokine ligand 25; CCR6, C-C chemokine receptor 6; CCR9, C-C chemokine receptor 9; CXCR3, C-X-C motif chemokine receptor 3; DLCO, diffusing capacity of the lung for carbon monoxide; FFAR2, free fatty acid receptor 2; FFAR3, free fatty acid receptor 3; HCAR2, hydroxycarboxylic acid receptor 2; HDAC, histone deacetylase; HRCT, high-resolution computed tomography; IBD, inflammatory bowel disease; IL-22, interleukin-22; LPS, lipopolysaccharide; MAdCAM-1, mucosal addressin cell adhesion molecule-1; SCFA, short-chain fatty acid; Th17, T helper 17 cell; TMA, trimethylamine; TMAO, trimethylamine N-oxide; Treg, regulatory T cell; TRM, tissue-resident memory T cell.

Although the aforementioned “microecology–metabolite–immunity” framework constitutes an important pathological basis for IBD-related pulmonary injury, the heterogeneity of clinical pulmonary phenotypes suggests that its occurrence is not determined solely by intrinsic disease processes; therapeutic interventions are also non-negligible influencing factors (6, 42, 123). Immunomodulators, biologics, and small-molecule targeted drugs suppress abnormal inflammatory responses but may also disrupt immune networks crucial for maintaining mucosal homeostasis, pathogen clearance, and host–microbe balance, thereby causing further clinical and radiological overlap of infectious and non-infectious pulmonary injury (124–126). Drug-related pulmonary injury in the context of IBD is better understood as a pulmonary manifestation jointly shaped by underlying immune imbalance, gut–lung axis dysregulation, and systemic pharmacologic intervention and should be included in clinical differential diagnoses alongside disease activity and infection risk.

## 5 Drug-related pulmonary injury in IBD: mechanisms and differential diagnosis

With the widespread use of immunomodulators and biologics in IBD, when respiratory symptoms or radiologic abnormalities appear in clinical practice, three major etiologies must be simultaneously considered: pulmonary EIM of the disease itself, infection (especially opportunistic infection), and treatment-related pulmonary injury. Pediatric patients with IBD warrant particular attention because pediatric-onset disease is often associated with a more extensive and severe clinical course than adult-onset disease, together with age-specific complications such as impaired growth and pubertal delay (127). Although pulmonary involvement in children with IBD appears to be uncommon and may be underrecognized, its clinical spectrum largely overlaps with that observed in adults and may include respiratory symptoms, airway inflammation, and reduced exercise tolerance (128). The latest European ECCO and Colitis Organization consensus on EIM also emphasizes treatment-related complications as an important component of IBD extraintestinal issues, underscoring that identifying and stratifying adverse drug reactions is critical to patient outcomes (123).

Drug-induced pulmonary injury (especially drug-induced interstitial lung disease/pneumonitis, DI-ILD/pneumonitis) is highly heterogeneous; its onset may be acute or insidious, clinical manifestations and imaging findings are often nonspecific, and there is significant overlap with infectious pneumonia, underlying interstitial lung disease, or IBD-related inflammatory pulmonary involvement, resulting in a high risk of missed or incorrect diagnoses. Authoritative reviews indicate that the diagnosis of DI-ILD typically relies on (1) a temporal association with exposure to the suspected drug (2); newly emerged or progressive parenchymal/interstitial pulmonary imaging changes; and (3) systematic exclusion of more common alternative diagnoses, highlighting that prompt discontinuation of the suspected drug is a critical step in improving outcomes (129, 130).

In the IBD population, several commonly used drugs have been reported to cause non-infectious pulmonary toxicity, including 5-aminosalicylic acid agents (e.g., mesalazine), immunomodulators (e.g., methotrexate), and certain biologics. For several forms of drug-related pulmonary injury, current evidence remains largely limited to case reports and small case series. Accordingly, etiologic attribution should be based on an integrated assessment of drug-exposure timing, radiological patterns, exclusion of infection, and response to drug withdrawal. For example, non-infectious pneumonia related to vedolizumab has been systematically described in case series (22), and mesalazine can also lead to interstitial or eosinophilic pneumonia, with a wide latency period and, in severe cases, respiratory failure; however, most patients improve after drug discontinuation and, when necessary, glucocorticoid therapy (131). For anti-TNF-α therapy, although large population safety studies suggest that it may not significantly increase the overall incidence of autoimmune ILD (132), clinicians should remain vigilant regarding rare reports of drug-related pneumonia/organizing pneumonia, and extra caution should be exercised in patients with underlying lung disease or an increased risk of infection (129, 130). Representative HRCT patterns, latency periods, key differential diagnoses, and management strategy recommendations for the above drug categories are summarized in **Table 2**.

**Table 2:** Differential diagnosis and initial management of drug-induced pulmonary toxicity in inflammatory bowel disease.

Drug class	Representative agents	Key pulmonary pattern/HRCT clues	Latency	Diagnostic priorities	Initial management	Reference
5-ASA/sulfasalazine	Sulfasalazine; mesalazine	Eosinophilic pneumonia, OP, or hypersensitivity pneumonitis; GGO ± consolidation; centrilobular nodules, mosaic attenuation/air trapping	Days–weeks; occasionally delayed	Prioritize infection and IBD-related pulmonary involvement; peripheral eosinophilia supports hypersensitivity	Immediate withdrawal; exclude infection; systemic corticosteroids if moderate–severe; avoid rechallenge	(6, 42, 123, 133)
Thiopurines	Azathioprine, 6-mercaptopurine	Interstitial pneumonitis/ILD, sometimes OP-like; GGO ± nodules or consolidation	Weeks–months, often early after initiation	PJP, CMV, fungal or mycobacterial infection; IBD-related lung disease	Withdraw drug; rule out infection first; consider corticosteroids if noninfectious toxicity is likely; avoid re-exposure	(6, 42, 123, 133)
Methotrexate	Methotrexate	Classic methotrexate pneumonitis/hypersensitivity-like pneumonitis; diffuse or patchy GGO, centrilobular nodules; NSIP- or OP-like pattern	Weeks–months, usually within first year	PJP, viral pneumonia, cardiogenic edema, or progression of pre-existing ILD	Immediate discontinuation; supportive care; corticosteroids for moderate–severe disease; do not rechallenge	(6, 42, 123, 133)
Anti-TNF agents	Infliximab; adalimumab; certolizumab; golimumab	ILD with OP- or NSIP-like features; occasional sarcoid-like granulomatous reaction with pulmonary nodules and hilar/mediastinal lymphadenopathy	Usually months; variable	Tuberculosis, nontuberculous mycobacterial or fungal infection, true sarcoidosis, and underlying ILD progression	Discontinue suspected agent; systematically exclude infection; corticosteroids after infection is reasonably excluded; switch therapeutic class if needed	(6, 42, 123, 133, 134)
Anti-integrin therapy	Vedolizumab	Noninfectious pneumonitis/ILD, often OP-like; GGO ± consolidation	Acute–subacute; usually weeks–months	Infection, IBD-related pulmonary disease, and clinicoradiologic improvement after withdrawal	Withdraw drug; corticosteroids for clinically significant cases; avoid rechallenge	(22, 123, 133)
Anti-IL-12/23 therapy	Ustekinumab	Rare noninfectious pneumonitis/ILD; GGO ± consolidation; occasional OP-like or eosinophilic pattern	Weeks–months	Infection, IBD-related pulmonary disease, autoimmune pneumonitis, and progression of baseline ILD	Discontinue drug; consider corticosteroids in moderate–severe cases; close follow-up with symptoms, imaging, and pulmonary function	(123, 133, 135)
JAK inhibitors	Tofacitinib; upadacitinib	Very rare suspected pneumonitis/ILD; GGO ± consolidation, occasionally OP-like	Weeks–months	Prioritize viral or opportunistic infection and pulmonary embolism	Withhold drug; evaluate infectious and thromboembolic causes first; consider corticosteroids only after major infection is excluded	(123, 133, 136)

HRCT, high-resolution computed tomography; GGO, ground-glass opacity; ILD, interstitial lung disease; OP, organizing pneumonia; NSIP, nonspecific interstitial pneumonia; PJP, *Pneumocystis jirovecii* pneumonia; CMV, cytomegalovirus.

Drug-induced pulmonary toxicity should be regarded as a diagnosis of exclusion. In immunosuppressed patients with inflammatory bowel disease, infectious etiologies should be systematically prioritized before pulmonary infiltrates are attributed to drug toxicity.

**Figure 4:**
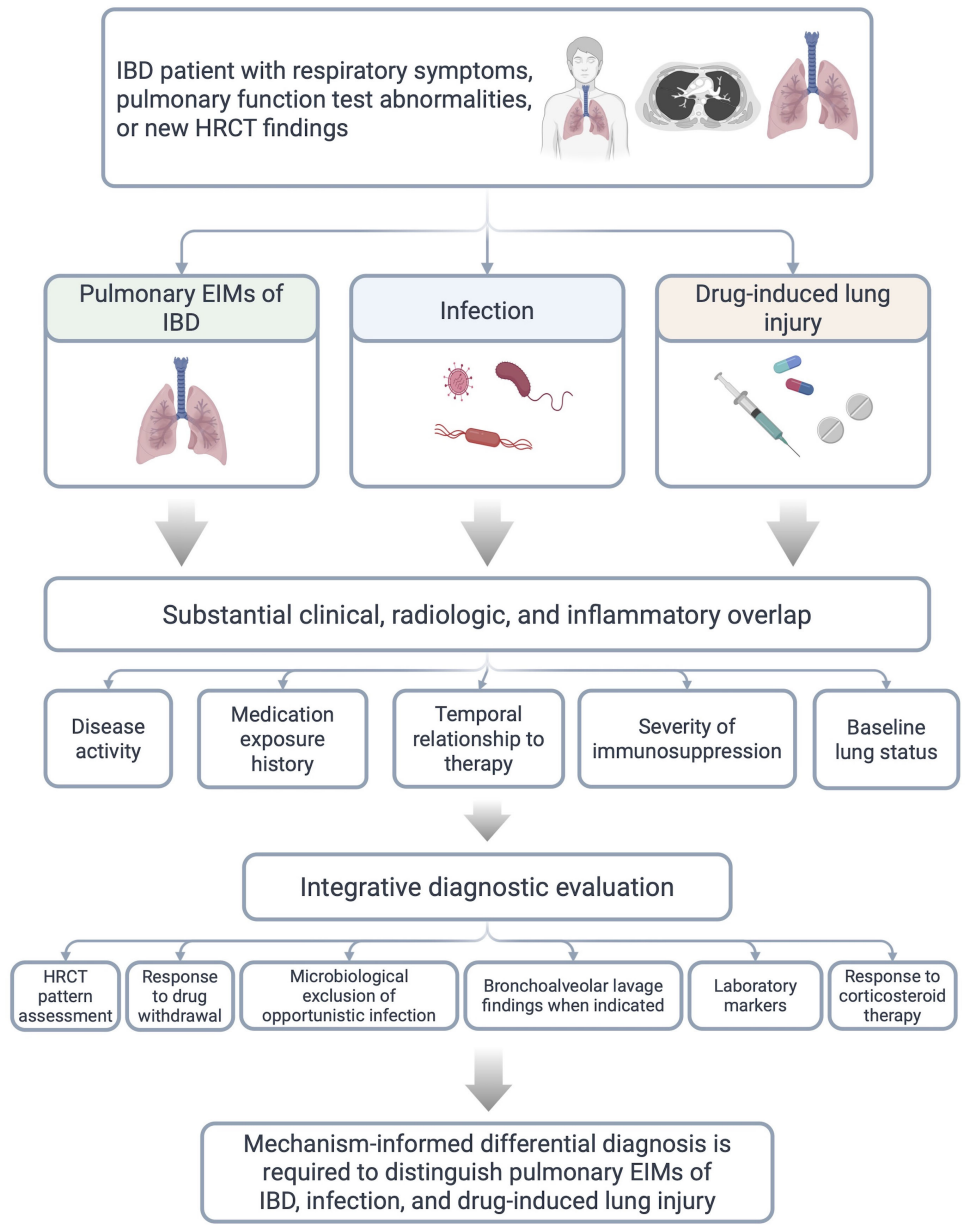
Diagnostic framework for pulmonary abnormalities in inflammatory bowel disease (IBD). In patients with IBD who develop respiratory symptoms, pulmonary function test abnormalities, or new high-resolution computed tomography (HRCT) findings, three major etiologies should be considered in parallel: pulmonary extraintestinal manifestations, infection, and drug-induced lung injury. Because these entities frequently share overlapping clinical and radiologic features, a differential diagnosis should integrate disease activity, medication exposure, temporal relationship to therapy, severity of immunosuppression, and baseline lung status. An integrative diagnostic evaluation includes HRCT pattern assessment, microbiological exclusion of opportunistic infection, bronchoalveolar lavage findings when indicated, laboratory markers, and dynamic response to drug withdrawal or corticosteroid therapy. This framework highlights the need for mechanism-informed and context-dependent interpretation of pulmonary abnormalities in IBD. Created in BioRender. Mu, Z. (2026) https://BioRender.com/gcsvy1e.

Given the considerable overlap among IBD-associated pulmonary EIMs, opportunistic infections, and drug-related lung injury in terms of clinical manifestations, HRCT patterns, and inflammatory markers, a single symptom or imaging feature is often insufficient for etiological attribution (25, 123, 129, 130). Based on a systematic review of drug-related lung injury, this article proposes a mechanism-oriented, stratified differential diagnostic algorithm that emphasizes a dynamic assessment that incorporates disease activity, timing of drug exposure, immunosuppression status, microbiological evaluation, and therapeutic response (**Figure 4**).

## 6 Translational opportunities and future directions

From a translational perspective, the microbiota–metabolite–immune axis provides a biologically plausible framework for developing mechanism-informed strategies for IBD-associated pulmonary injury (19, 21, 137). Evidence from studies of probiotics, dietary optimization, and fecal microbiota transplantation (FMT) in IBD, particularly ulcerative colitis, suggests a signal of clinical translatability in restoring microbial homeostasis and modulating mucosal inflammation (138–140). However, current guidelines still recommend that conventional FMT for ulcerative colitis and Crohn’s disease should be restricted to clinical trial settings, underscoring the need for disease-specific efficacy and safety validation (139). Moreover, evidence directly linking microbiota–metabolite–immune interventions to IBD-associated pulmonary complications remains limited and is largely derived from mechanistic studies (19, 21, 137). Accordingly, prospective studies are needed to identify suitable target populations, determine optimal intervention windows, validate microbial and metabolic response markers, and establish safety boundaries. In parallel, increasing evidence of a resident respiratory microbiota supports the integration of pulmonary microbiome alterations into a broader bidirectional gut–lung axis framework (141, 142).

Future research should prioritize the development of reproducible and clinically translatable biomarker systems that can distinguish drug-induced lung injury from disease-related pulmonary involvement and infection. Given the absence of pathognomonic clinical, radiological, or pathological features in DI-ILD, integrated models incorporating drug exposure history, temporal association, microbiological exclusion, radiomics, peripheral blood or bronchoalveolar lavage multi-omics, and clinical treatment trajectories may improve etiologic attribution and risk prediction (129, 143). In addition, the therapeutic and prophylactic value of gut microbiota modulation for IBD-associated lung injury should be evaluated using both mechanistic endpoints, such as SCFA levels, immune-cell trafficking, immune differentiation, and barrier markers, and clinical endpoints, including lung function, HRCT-based quantification, and exacerbation risk (19, 21, 137, 141). Collectively, IBD-associated lung injury may be conceptualized as an interconnected pathological cascade linking gut microbial dysbiosis, metabolic disturbances, systemic immune remodeling, and heightened pulmonary susceptibility, providing a basis for future precision diagnosis, patient stratification, biomarker development, and individualized therapeutic intervention.

## 7 Conclusion

Pulmonary injury in IBD should not be regarded simply as an isolated extraintestinal manifestation. Rather, it may represent a clinically heterogeneous outcome of systemic mucosal immune dysregulation, in which intestinal barrier failure, microbiota dysbiosis, aberrant immune trafficking, and treatment-related perturbation act together to shape pulmonary involvement. Accumulating evidence suggests that intestinal barrier disruption, microbial translocation, dysbiosis-derived metabolite imbalance, aberrant immune-cell trafficking, and systemic inflammatory dissemination collectively contribute to pulmonary immune remodeling and lung injury in susceptible individuals. Importantly, therapeutic interventions may further modify this process by altering host defense, mucosal homeostasis, and immune surveillance, thereby creating substantial overlap between inflammatory, infectious, and drug-induced pulmonary phenotypes.

Pulmonary involvement in IBD likely emerges from the convergence of epithelial dysfunction, microbiota-dependent signaling, systemic immune activation, and treatment-related perturbation within a shared mucosal immune network, rather than being driven by a single pathogenic mechanism. This integrative framework may explain the marked heterogeneity of pulmonary manifestations observed across clinical, radiologic, and pathologic settings and provide a conceptual basis for mechanism-informed diagnostic and therapeutic strategies.

Despite growing recognition of gut–lung crosstalk, major gaps remain regarding causal pathways, temporal dynamics, disease susceptibility, and biomarker identification. Continued integration of mucosal immunology, microbiome science, multi-omics profiling, and longitudinal clinical phenotyping will likely be essential for refining disease stratification and improving the early recognition of pulmonary complications in IBD. Ultimately, a deeper mechanistic understanding of the gut–lung axis may not only improve the management of pulmonary involvement in IBD but also advance broader insights into systemic immune-mediated inflammatory diseases.
